# Air Temperature Change in the Southern Tarim River Basin, China, 1964–2011

**DOI:** 10.1155/2013/894851

**Published:** 2013-11-19

**Authors:** Benfu Zhao, Jianhua Xu, Zhongsheng Chen, Ling Bai, Peng Li

**Affiliations:** ^1^The Research Center for East-West Cooperation in China, The Key Laboratory of Geographic Information Science, The Ministry of Education, East China Normal University, Shanghai 200241, China; ^2^State Key Laboratory of Desert and Oasis Ecology, Xinjiang Institute of Ecology and Geography, Chinese Academy of Sciences, Urumqi 830011, China

## Abstract

The temperature data from 3 meteorological stations (Kashi, Ruoqiang, and Hotan) in the South of Tarim River Basin (STRB) during 1964–2011 were analyzed by Mann-Kendall test and correlation analysis. The results from Mann-Kendall test show that the surface temperature (ST), 850 hPa temperature (T850), and 700 hPa temperature (T700) exhibited upward trends, while 300 hPa temperature (T300) revealed a downward trend. On the whole, the change rate of ST, T850, T700, and T300 was 0.26*~*0.46°C/10a, 0.15*~*0.40°C/10a, 0.03*~*0.10°C/10a, and −0.38*~*−0.13°C/10a, respectively. For the periods, ST and T850 declined during 1964–1997 and then rose during 1998–2011. T700 declined during 1964–2005 and then rose during 2006–2011, while T300 rose from 1964 to 1970s and then declined. The results from correlation analysis show that T850 and T700 positively correlated with ST (*P* < 0.01) at the all three stations and there was a negative correlation between T300 and ST at Hotan (*P* < 0.1), while the correlation is not significant at Kashi and Ruoqiang. The results indicate that there were gradient differences in the response of upper-air temperature (UT) to ST change.

## 1. Introduction

With the rapid development of global social economy, diversified human activities have significant influences on global climate system. The fourth IPCC Report shows that the latest 100-year linear trend (1906 to 2005) of 0.74°C is therefore larger than the corresponding trend for 1901 to 2000 given in the TAR of 0.6°C. Eleven of the last twelve years (1995–2006) rank among the 12 warmest years in the instrumental record of global surface temperature since 1850 [1]. The troposphere and stratosphere are important parts of climate system, and the determination on the trends of upper-air temperature (UT) has been an indispensable foundation for climate change research. UT's trends are strongly connected to the problem of global warming [2, 3], but their patterns are somewhat different from those on the land surface [4] and carry a large uncertainty. Therefore, the patterns of long-term trends in upper-air temperature series have become the focus of numerous discussions in recent years [5–9]. Ren et al. [10] find that annual mean ST in Chinese mainland as a whole rose by about 1.1°C for the last 50 years, with a warming rate of about 0.22°C/10a, based on national reference climatological stations and basic meteorological ground station data. Chen et al. [11] used the monthly mean temperature data of 19 meteorological stations from 1961 to 2008 in the Yili River Valley, analyzed the correlation between mean annual temperature and elevation, and obtained the temperature lapse rate of 0.564°C/100 m. The results reflected the spatial variability of temperature. Guo and Ding [12] analyze the change trend of high atmosphere temperature in China from 1958 to 2005 using the radiosonde sounding data of China's 116 sounding stations and find that the high atmosphere temperature below 400 hPa standard barosphere showed a significant upward trend with the amplitude particularly prominent in the high-altitude areas. Free and Seidel [8] find the temperature from ground to 300 hPa all warming based on LKS radiosonde data, but cooling based on HadRT data.

The arid area of Northwest China and the Tibetan Plateau are sensitive areas of climate change; many researchers have launched a lot of research and discussion about them [13–18]. These pieces of research include ST and UT change, but the research about the relation of ST and UT are very few. This paper studies the relationship between ST and UT and tries to establish the relationship between ST and UT for the quantitative evaluation of human activities on climate change. 

**Figure 1:**
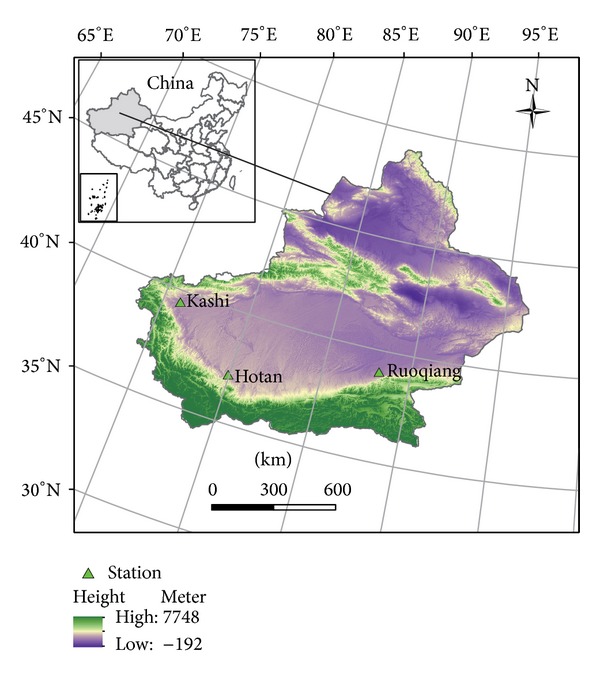
The sketch map of study area in Xinjiang, China.

Qinghai-Tibet Plateau is an active and important area for stratosphere-troposphere exchange. The study area of this paper is very special, located in the southern edge of the Tarim River Basin, and although the three meteorological stations (Kashi, Ruoqiang, and Hotan) are located in the arid area of northwest, their locations are very close to the Qinghai-Tibet Plateau, as given in Figure 1. The temperatures in this region may be affected by the climate of the Qinghai-Tibet plateau and the northwest arid areas. Quantitative research temperature changes of the study area may provide a new insight into the understanding of UT and ST at a climatic edge.

## 2. Data and Methods 

### 2.1. Study Area

The Tarim River basin with the area of 1,020,000 km^2^ covers the entire south Xinjiang province in China (Figure 1). Its area is 1.4 times the Yellow River basin, and it is populated with 8,257,000. The mainstream catchment of the Tarim River basin, with the length of 1,321 km, an area of 17,600 km^2^, and a population of 120,100, is located in the extreme arid region receiving an annual rainfall of less than 50 mm with the potential evaporation of more than 2,000 mm. In the past 50 years, the temperature of Xinjiang is rising, the average increase is 0.27°C/10a, and the northern region is 0.36°C/10a, the southern region is 0.2°C/10a [13].

### 2.2. Data

The data used in this study were from three meteorological stations, located in the south of Tarim River basin, and their locations and elevations are shown in Table 1. Considering completeness and comparability of the data, this paper selected the monthly temperature data from 1964 to 2011. Missing data for individual months were replaced with the average value of the same month data in adjacent two years. The temperature data of 850–300 hPa are radiosonde data. All data were tested for homogeneity and corrected.

### 2.3. Methods

#### 2.3.1. Trend Test

Nonparametric test is not affected by the data distribution, so it has wider application range. Nonparametric test treatment methods are based on low-precision data, so that they can handle almost any types of data. Mann-Kendall (MK) method is a nonparametric statistical test.

The MK nonparametric trend test is commonly used to assess the significance of monotonic trends in meteorological and hydrologic series all over the world [19, 20]. For a time series *X* = {*x*_1_, *x*_2_ … *x*_*n*_}, in which *n* > 10, the standard normal statistic *Z* is estimated as

(1) $$\begin{array}{c} Z_{c}=\{\begin{array}{cc} \frac{S-1}{\sqrt{\text{var}\left(S\right)}}, & S>0\\ 0, & S=0\\ \frac{S+1}{\sqrt{\text{var}\left(S\right)}}, & S<0, \end{array} \end{array}$$

where

(2) $$\begin{array}{c} S=\overset{n-1}{\underset{i=1}{\sum }}\overset{n}{\underset{k=i+1}{\sum }}\mathrm{sgn}\left(x_{k}-x_{i}\right),\\ \mathrm{sgn}\left(\theta \right)=\{\begin{array}{cc} 1, & \theta >0\\ 0, & \theta =0\\ -1, & \theta <0, \end{array}\\ \text{var}\left[S\right]=\frac{\left[n\left(n-1\right)\left(2n+5\right)-\sum_{t}^{}t\left(t-1\right)\left(2t+5\right)\right]}{18}. \end{array}$$

The statistic *Z* follows the standard normal distribution. At a 5% significance level, the null hypothesis of no trend is rejected if |*Z*| > 1.96. A positive value of *Z* denotes an increasing trend, and the opposite corresponds to a decreasing trend.

#### 2.3.2. Abrupt Change Point Analysis

The MK nonparametric test is widely applied for determining the occurrence of abrupt change points of meteorological and hydrologic series. Advantage of the method is not only simple calculation but also confirmation of the starting time of abrupt changes and identification of the area of abrupt changes [21]. Let *x*_1_ ⋯ *x*_*n*_ be the data points. For each element *x*_*i*_, the numbers *r*_*i*_ of elements *x*_*j*_ preceding it (*j* < *i*) such that *x*_*j*_ < *x*_*i*_ are computed. Under the null hypothesis (no abrupt change point), the normally distributed statistic *S*_*k*_ can be calculated via the following formula:

(3) $$\begin{array}{c} S_{k}=\overset{k}{\underset{j=1}{\sum }}r_{i},\;2\leq k\leq n. \end{array}$$

Mean and variance of the normally distributed statistic *S*_*k*_ can be given by the following formulas:

(4) $$\begin{array}{c} S=E\left(S_{k}\right)=\frac{k\left(k-1\right)}{4},\\ \text{ var }\left(S_{k}\right)=\frac{k\left(k-1\right)\left(2k+5\right)}{72}. \end{array}$$

The normalized variable statistic UF_*k*_ is estimated as follows:

(5) $$\begin{array}{c} \text{U}{\text{F}}_{k}=\left(S_{k}-S\right)\sqrt{\text{var}\left(S_{k}\right)}. \end{array}$$

The normalized variable statistic UF_*k*_ is the forward sequence, and the backward sequence UB_*k*_ is calculated using the same equation but with a reversed series of data. When the null hypothesis is rejected (i.e., if any of the points in the forward sequence is outside the confidence interval), the detection of an increasing (UF_*k*_ > 0) or a decreasing (UF_*k*_ < 0) trend is indicated. The sequential version of the test used here enables detection of the approximate time of occurrence of the trend by locating the intersection of the forward and backward curves of the test statistic. If any intersection appears in the confidence interval, it indicates an abrupt change point.

## 3. Results and Discussions

### 3.1. Change Trends

ST and T300 exhibited the opposite change trend; ST showed an upward trend, while T300 presented a downward trend. The *Z* values declined with height, and the changes in amplitude first decrease and then increase. This shows that the temperature trend was reversed between the ground and 300 hPa. The research of Xue et al. [22] had confirmed the opposite change trend of ST and UT. The observed pattern of tropospheric warming and stratospheric cooling is very likely due to the combined influences of greenhouse gas (GHG) increases and stratospheric ozone depletion [1].

**Figure 2:**
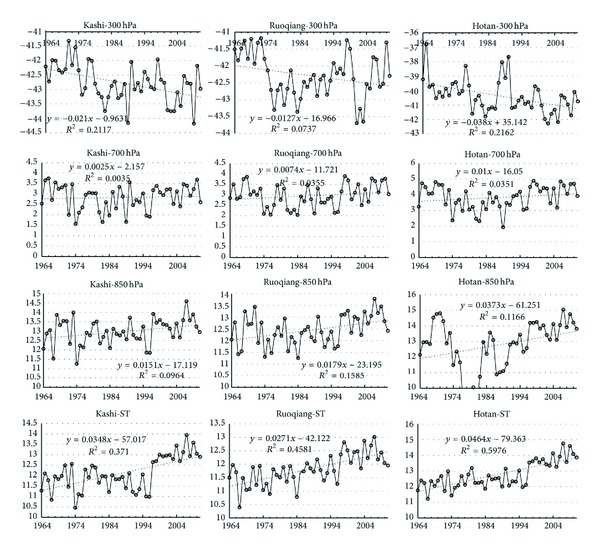
The temperature change of three stations.

ST was lower than T850 before 1974, but ST overtakes T850 after 1974. In addition to T300, minimum temperature or subminimum temperature of the other heights occurred in 1974–1979.  Figure 2 and Table 2 show that Hotan's temperature variation is the largest, Ruoqiang's was the smallest, and Kashi's was in the middle.

Three weather stations, ST, T850, and T700, had the same change trend, but the ascending range of ST was larger than T850, and T850 was larger than T700. The possible reasons of the results are as follows.

With the increase of greenhouse gases, it is no doubt that ST is significantly increased due to the greenhouse effect. In the troposphere, the increasing greenhouse gases can absorb more long-wave radiation from the ground, which will make tropospheric temperature rise. However, as more long-wave radiation absorbed by the troposphere, the stratosphere will receive less long-wave radiation. And meanwhile, the increasing greenhouse gases also transport more heat to the cosmic space in the form of infrared radiation energy. Consequently, the stratospheric temperature will drop [23].

### 3.2. Abrupt Change Point

**Figure 3:**
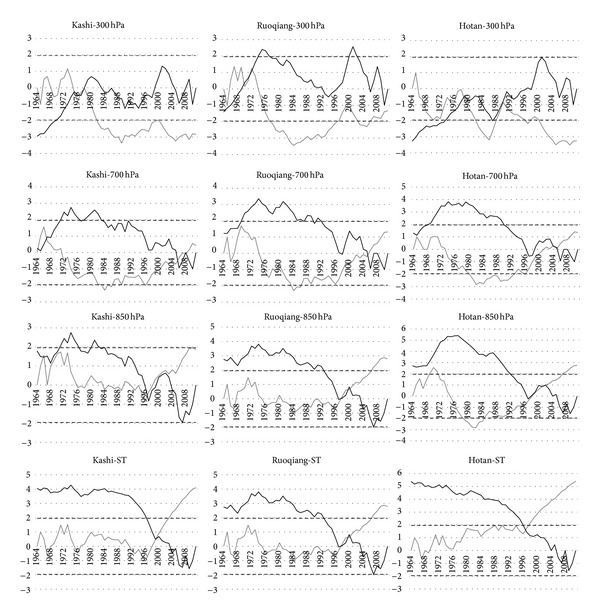
Abrupt change point analysis of three stations.

Figure 3 shows that the abrupt change of ST occurred in the late 1990s except for Ruoqiang in the early 1990s (Table 2), while Xue and Chen believe that the abrupt change of ST in Northwest China occurred in the early 1990s [24]; the abrupt changes of T850 all occurred around 1997, while the abrupt changes of T700 which occurred around 2005 did not pass the significance test; the abrupt changes of T300 occurred in the late 1970s. The northern hemisphere temperature abrupt change occurred in 1988 [25], and however, all temperatures' abrupt changes occurred later than 1988 except for T300. The results showed that the height of any temperature mutation time changes with place, namely, the mutation points of temperature largely dominated by regional factors.

**Figure 4:**
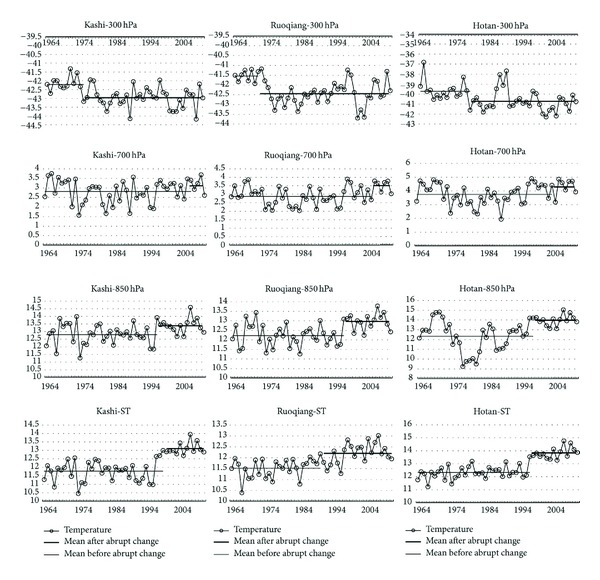
The difference in mean temperature of abrupt change before and after.

Figure 4 shows that each height's temperature in three stations has the same trend and similar abrupt changes. The average of all temperatures before mutation is less than after mutation except T300. T300 has the largest temperature difference before and after the mutation. These results suggest that ST, T850, and T700 may belong to an atmospheric layer, while T300 may belong to another atmospheric layer. This may suggest that the tropopause height of STRB lower than same latitude areas.

### 3.3. Correlation Analysis

The Pearson correlation analysis shows that T850 and T700 have more significantly positive relationships (significant at 1% level) with ST among all stations in STRB than T300 (shown in Table 3), which indicates that T850 and T700 have closer relations with ST than T300 in the STRB. T850 and T700 were positively correlated with ST, significant at 1% levels in all stations, while the correlation coefficient between ST and T300 reaches a weak significance level. Compared to the other two stations, Hotan showed a difference that T300 was negatively correlated with ST in Hotan, significant at 10% level.

Based on the above analysis, the regression equations of ST and UT were established, as given in Table 4. Table 4 shows that the correlation coefficients of ST and UT among all stations in STRB were decreased with increase in height. T850 and T700 showed a strongly positive correlation with ST, but T300 showed a weakly negative correlation with ST.

For Ruoqiang and Kashi stations, changes in tropospheric temperature show significant gradient effect due to ground radiation, namely, the standard pressure levels farther away from the ground; the correlation between UT change and ST change becomes weaker. And 300 hPa belong stratosphere (above the troposphere) are almost not sensitive to ground radiation, solar radiation is its direct heat. The decrease of T300 may be affected by solar radiation and ozone changes. Hotan is located in the northwest edge of Qinghai-Tibet plateau with complex terrain, and its conditions are remarkably affected by surrounding. The special geographic characteristics inevitably result in particular local circulation, which disturbs the gradient effect of tropospheric temperature change to some extent. T300 and ST have negative relationships at 10% significance level, which only have statistical significance but not practical significance.

This indicates the lower stratosphere and troposphere are independent of each other and belong to different atmospheric layers; that is, changes in ST have little effect on temperature changes of the atmosphere above the troposphere.

### 3.4. Discussion

Through the above analysis, we found two interesting results. Firstly, the atmosphere temperatures below the height of 700 hPa exhibit increasing trend, while T300 shows an opposite trend, and secondly, there are spatial differences in the change rates and change points of ST and UT. 

There are many factors causing this regional difference [26]. Firstly, the three stations are located in different geographical conditions which will affect long wave radiation from surface to upper air; secondly, the way and intensity of human activities are different in the three areas, and meanwhile, land use/cover is also distinct, so greenhouse gases and aerosols from human activities will contribute to the unique temperature change in the locality; thirdly, due to different geographical conditions, each area can form the unique local atmospheric circulation affecting temperature change to some extent. Moreover, the Tibetan plateau is a huge heat source, and it can heat the middle and upper atmosphere above the plateau and the surrounding areas by releasing heat flux [27–29]. Hotan is located in the northwest part of the Tibetan plateau, and its middle and upper atmosphere are notably affected by the heat flux from the Tibetan plateau. So in Hotan, T700 has a closer relation with ST than T850, which is different from the other two stations and does not comply with the general rules either.

## 4. Conclusion

Through the above analysis of the time series change trends of UT and ST, and their abrupt change in the Southern Tarim River Basin during the period 1964–2011, the main conclusions of this paper are drawn as follows.

1. In the past 48 years, ST, T850, and T700 exhibit an upward trend, but T300 shows a downtrend in STRB. Although T700 exhibits an increasing trend, the change of T700 does not pass the 10% level of significance test. There are obvious differences in the change of ST, T850, T700, and T300 in both horizontal space and vertical space.
2. Abrupt change points of T300 and T700 occurred in the 1970s and 2005, respectively. However, abrupt change points of T850 and ST both occurred in 1990s. The abrupt change of temperature in all heights occurred later than that of the northern hemisphere temperature (1988) except for T300.
3. With the increase of height, the correlation between ST and UT heights temperature was decreasing except Hotan, and the correlation between ST and T300 approximates to zero. T850 and T700 were positively correlated with ST at 1% significance level, while T300 was negatively correlated with ST and the correlation coefficient was not significant.

## Figures and Tables

**Table 1:** The stations of the Southern Tarim River Basin.

Station name	Landscapes	Longitude (/°E)	Latitude (/°N)	Elevation (/m)
Kashi	Plain	75.98	39.47	1,291
Hotan	Plain	79.93	37.13	1,375
Ruoqiang	Basin	88.17	39.03	889

**Table 2:** The test results of change trends and abrupt changes of the three stations.

Temperature	Trend test			Abrupt change points
	Trend rate (°C/10a)	*Z* values	Significance level	
Kashi station				
ST	0.34	4.03	0.05	1999
T850	0.15	1.7954	—	1997
T700	0.025	0.32	—	2007
T300	−0.21	−2.8975	0.05	1975

Ruoqiang station				
ST	0.27	4.817	0.05	1990
T850	0.18	2.7553	0.05	1997
T700	0.07	1.2443	—	2005
T300	−0.12	−1.3332	—	1971

Hotan station				
ST	0.46	5.39	0.05	1997
T850	0.37	2.7197	0.05	1998
T700	0.10	1.3154	—	2004
T300	−0.38	−3.2708	0.05	1979

**Table 3:** Correlation analysis of ST and UT.

Pearson correlation			
Stations	Kashi	Ruoqiang	Hotan
ST and T850	0.812**	0.752**	0.480**
ST and T700	0.602**	0.532**	0.529**
ST and T300	−0.142	−0.025	−0.268*

Note: **correlation is significant at the 0.01 level (2-tailed); *correlation is significant at the 0.1 level (2-tailed).

**Table 4:** Correlation analysis of ST and UT.

Regression equation						
Station	Kashi	*R* ^2^	Ruoqiang	R^2^	Hotan	R^2^
ST & T850	*y*_1_ = 0.69*x* + 4.59^a^	0.66	*y*_1_ = 0.84*x* + 2.44^a^	0.57	*y*_1_ = 0.87*x* + 1.70^a^	0.23
ST & T700	*y*_2_ = 0.45*x* − 2.57^a^	0.37	*y*_2_ = 0.53*x* − 3.28^a^	0.29	*y*_2_ = 0.47*x* − 2.20^a^	0.28
ST & T300	*y*_3_ = −0.12*x* − 41.37	0.02	*y*_3_ = −0.02*x* − 42.04	0.00	*y*_3_ = −0.37*x* − 35.63^b^	0.07

Note: *x*-ST, *y*_1_-T850, *y*_2_-T700, *y*_3_-T300; ^a^correlation is significant at the 0.01 level; ^b^correlation is significant at the 0.1 level.
